# Integrating Pharmacovigilance Data Mining and Mendelian Randomization to Identify Risk Profiles and Causal Targets of Opioid‐Induced Delirium

**DOI:** 10.1002/cns.70983

**Published:** 2026-06-17

**Authors:** Xuetai Chen, Yuting Liu, Mingxuan Zhu, Xingman Chen, Fengyun Liu, Jiahao Jin, Zhiguo Jiang, Bin Qian, Feng Li

**Affiliations:** ^1^ Department of Anesthesiology The Yancheng Clinical College of Xuzhou Medical University, the First People's Hospital of Yancheng Yancheng China; ^2^ Jiangsu Province Key Laboratory of Anesthesiology Xuzhou Medical University Xuzhou China

**Keywords:** delirium, FAERS, Mendelian randomization, network pharmacology, opioids, pharmacovigilance

## Abstract

**Background:**

Opioid‐induced delirium represents a major clinical challenge with substantial implications for patient safety and outcomes. An integrated evaluation combining pharmacovigilance and genetic evidence remains limited. This study aimed to systematically assess delirium risk across different opioids and to elucidate underlying molecular mechanisms by integrating real‐world safety data with Mendelian randomization (MR) analysis.

**Methods:**

Using data from the US Food and Drug Administration Adverse Event Reporting System (FAERS) spanning 2004–2025, disproportionality analyses (reporting odds ratio, proportional reporting ratio, and empirical Bayes geometric mean) and multivariable logistic regression were conducted to quantify delirium risk associated with 12 commonly used opioids. Network pharmacology was subsequently applied to identify candidate pathways and targets, followed by MR analyses based on FinnGen data to investigate potential causal relationships between key target genes and delirium.

**Results:**

Substantial heterogeneity in delirium risk was observed across opioids. Methadone showed the strongest association with delirium (EBGM 25.98) and the highest independent risk after adjustment (adjusted odds ratio 29.79), followed by oxycodone. Stronger risk signals were consistently observed among older individuals (> 55 years) and females. Mechanistic analyses highlighted pathways related to xenobiotic metabolism and neuroinflammation, particularly the PI3K‐Akt signaling pathway. MR analyses identified *COMT* as a risk‐associated gene for delirium (OR 1.25), whereas *MYD88* and *CYP1B1* demonstrated potential protective effects.

**Conclusions:**

Marked differences in delirium risk exist among opioids. *COMT*, *MYD88*, and *CYP1B1* were identified as key candidate genes underlying opioid‐associated delirium. These findings provide a genetic and mechanistic framework to support clinical risk stratification and the development of precision prevention and intervention strategies.

## Graphical abstract

Opioids like methadone and oxycodone pose a high risk for delirium, particularly in older adults and females. By integrating pharmacovigilance data and Mendelian randomization, we identify key genes (COMT, MYD88, CYP1B1) that influence delirium risk. These findings provide a genetic and mechanistic framework to guide opioid selection and delirium prevention strategies.

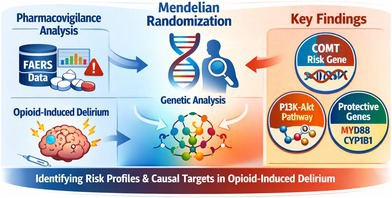

## 1 Introduction

Delirium is an acute state of cerebral dysfunction, typically characterized by impaired consciousness, attentional deficits, and cognitive disturbance [1]. It is highly prevalent among hospitalized patients, particularly in the perioperative setting and intensive care units, where reported incidence rates exceed 50% and 70%, respectively [2]. Clinically, delirium manifests as fluctuating disturbances in consciousness, cognition, and behavior, with episodes lasting from hours to days and a markedly variable course [3].

Opioids remain a cornerstone of multimodal analgesia in perioperative care. At a national level, it is estimated that 5.9%–6.5% of patients develop new persistent opioid use following surgery [4]. With the widespread implementation of Enhanced Recovery After Surgery (ERAS) protocols, intraoperative and postoperative opioid exposure has been substantially reduced [5, 6]. However, the incidence of opioid‐associated delirium has not declined in parallel and remains high, particularly among older adults and patients undergoing major surgical procedures. Moreover, opioid‐associated delirium has been independently linked to long‐term postoperative cognitive decline [7]. Despite its clinical significance, there is currently a lack of practical predictive models and effective interventional targets, substantially limiting precision prevention and treatment of opioid‐associated delirium.

Since its public release in 2004, the US Food and Drug Administration Adverse Event Reporting System (FAERS) has accumulated more than 15 million spontaneous reports encompassing drugs, biologics, vaccines, and medical devices [8]. Owing to its large scale, extended time span, and multidimensional data structure, FAERS has become a valuable resource for detecting drug–outcome association signals [9]. Mendelian randomization (MR), which uses genetic variants as instrumental variables to infer causal relationships between exposures and outcomes, can effectively mitigate confounding and reverse causation, and has emerged as an important approach for causal inference in drug target and disease research [10].

In this study, we adopted an integrated strategy combining pharmacovigilance signal detection, mechanistic pathway analysis, and genetic causal inference to systematically investigate the association between opioid use and delirium. By linking large‐scale FAERS data with network‐based biological analyses and Mendelian randomization, we aimed to characterize drug‐specific delirium risk profiles and identify key molecular pathways underlying opioid‐associated delirium. These findings are intended to inform precision risk stratification and support the development of targeted strategies for delirium prevention and optimization of perioperative analgesic management.

## 2 Methods

### 2.1 Data Sources and Study Design

A retrospective pharmacovigilance study design was adopted using the US Food and Drug Administration Adverse Event Reporting System (FAERS), a large, publicly accessible spontaneous reporting database widely used for post‐marketing drug safety surveillance in pharmacoepidemiological research [11]. FAERS collects individual case safety reports submitted by health‐care professionals, consumers, and pharmaceutical manufacturers, providing detailed information on suspected adverse drug reactions observed in real‐world clinical practice.

All publicly available FAERS data from January 1, 2004, to June 30, 2025, were integrated for analysis. Data extraction encompassed seven standardized FAERS datasets, including demographics, drug information, adverse reactions, outcomes, report sources, therapy dates, and indications, to construct a comprehensive drug‐adverse event analytical framework.

Standardized data preprocessing and integration were performed in accordance with FDA‐recommended procedures. CaseID served as the primary identifier, with auxiliary variables including sex, age, and event date used for record‐level cross‐validation. Duplicate reports arising from follow‐up submissions or multiple filings were systematically identified and removed to minimize statistical redundancy and preserve sample independence.

### 2.2 Definition of Adverse Events and Drug Exposure

Adverse event terminology was standardized using the Medical Dictionary for Regulatory Activities (MedDRA) [12]. Delirium was identified through the corresponding MedDRA Standardized MedDRA Query (SMQ) using a narrow‐scope strategy to maximize clinical specificity. Under this approach, only Preferred Terms with a high likelihood of representing true delirium cases were retained, thereby minimizing misclassification arising from nonspecific neuropsychiatric symptoms [13]. Included terms were restricted to those capturing the core clinical manifestations of delirium, such as “delirium,” “postoperative delirium,” and “delirium tremens,” while broader or potentially ambiguous cognitive or behavioral terms were deliberately excluded [14] (Table S1).

**FIGURE 1:**
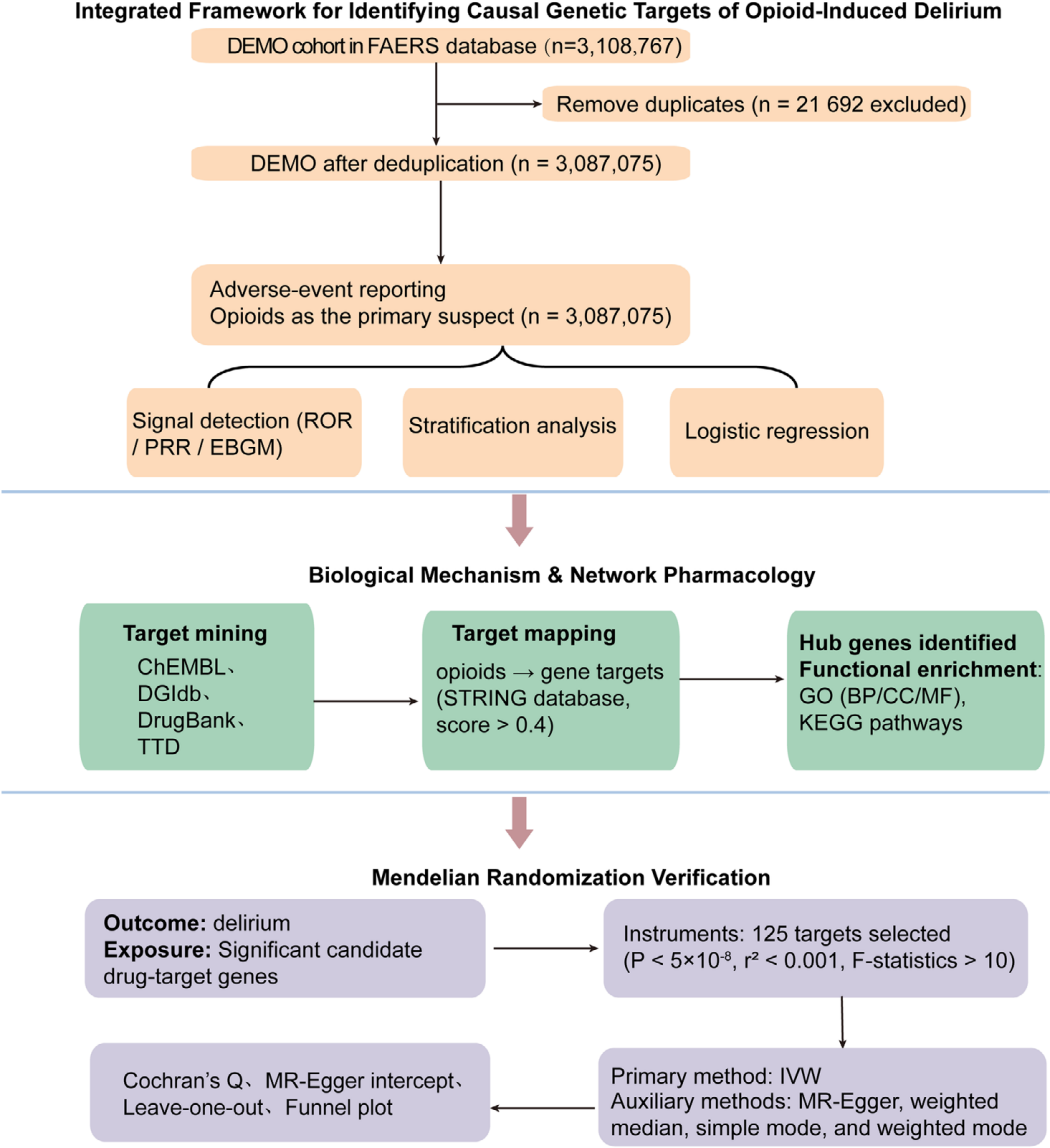
Flow chart.

Drug exposure was restricted to 12 commonly prescribed opioid and adjunct analgesic or sedative agents, including fentanyl, remifentanil, dexmedetomidine, morphine, methadone, tramadol, oxycodone, hydromorphone, codeine, hydrocodone, buprenorphine, and sufentanil (Figure 1). To mitigate confounding from polypharmacy, only reports in which target drugs were classified as Primary Suspect or Secondary Suspect were retained for analysis.

### 2.3 Disproportionality Signal Detection

Associations between target drugs and delirium were evaluated using disproportionality analysis integrating both frequentist and Bayesian approaches. Reporting Odds Ratio (ROR), Proportional Reporting Ratio (PRR), and Empirical Bayes Geometric Mean (EBGM) were calculated based on two‐by‐two contingency tables contrasting co‐reporting frequencies of drug‐delirium pairs against all other drug‐event combinations [15] (Table S2).

ROR quantified the relative reporting odds of delirium for a given drug compared with all other adverse events, whereas PRR assessed the proportional concentration of delirium reports among exposed vs. non‐exposed drugs. EBGM was introduced as a Bayesian shrinkage estimator, comparing observed co‐occurrence counts with expected frequencies derived from background reporting rates under prior constraints. This approach attenuated instability and false‐positive inflation associated with sparse data or low‐frequency reporting.

To enhance signal robustness and specificity, drug‐event associations were required to meet positive thresholds in at least two independent algorithms to qualify as statistically significant signals (Table S3). EBGM estimates were subsequently used as the primary metric of association strength, with heatmaps generated to visualize risk gradients across different opioid agents. Subgroup analyses stratified by age (≤ 55 years vs. > 55 years) and sex were conducted to explore demographic heterogeneity and potential effect modification.

### 2.4 Multivariable Regression Analysis

Given the inherent limitations of spontaneous reporting systems, including reporting bias and residual confounding, multivariable logistic regression models were constructed to estimate the independent association between drug exposure and delirium [16]. Delirium occurrence was modeled as a binary outcome, with covariates including age, sex, and ten drugs demonstrating positive disproportionality signals. Adjusted odds ratios (OR) with corresponding 95% confidence intervals (CI) were calculated to quantify associations after demographic adjustment.

### 2.5 Network Pharmacology Analysis

Based on high‐risk opioid agents identified through pharmacovigilance analyses, network pharmacology investigations were conducted to explore potential mechanistic pathways. Official drug target information was systematically retrieved from DrugBank, ChEMBL, DGIdb, and the Therapeutic Target Database (TTD). To enable subsequent Mendelian randomization (MR) analyses, opioid‐related candidate drug targets identified as significant were individually mapped to their corresponding genes and then merged to generate a unified “opioid‐delirium” drug target gene set [17].

Functional enrichment analyses were performed using the R package clusterProfiler (version 4.16.0), encompassing Kyoto Encyclopedia of Genes and Genomes (KEGG) pathways and Gene Ontology (GO) categories, including biological processes, cellular components, and molecular functions [18]. Adjusted *p*‐values below 0.05 were considered statistically significant. Shared targets were imported into the STRING database with a protein–protein interaction (PPI) confidence threshold of 0.4, and interaction networks were visualized using Cytoscape to identify key regulatory nodes.

### 2.6 MR Analysis

To evaluate potential causal relationships between core drug targets and delirium at the genetic level, MR analyses were performed. Core genes identified through network pharmacology were used as exposure variables, whereas delirium data were derived from the FinnGen biobank, comprising 210,756 individuals of European ancestry (finn‐b‐F5_DELIRIUM) [19]. Outcomes encompassed delirium associated with dementia, postoperative delirium, and other unspecified delirium phenotypes. Delirium subtypes attributable to alcohol or other psychoactive substances were excluded.

Instrumental variables were selected in strict accordance with the MR assumptions. Single‐nucleotide polymorphisms (SNPs) reaching genome‐wide significance (*p* < 5 × 10^−8^) were retained, with weak instruments excluded based on F‐statistics below 10 [20]. Linkage disequilibrium pruning was conducted using the European reference panel from the 1000 Genomes Project (window size 1,000 kb, r^2^ < 0.001) to ensure independence. Effect alleles were harmonized across exposure and outcome datasets, with palindromic variants and allele frequency mismatches removed.

Causal estimates were primarily derived using the inverse variance weighted (IVW) method, complemented by MR‐Egger regression, weighted median, simple mode, and weighted mode approaches for robustness assessment. Heterogeneity was evaluated using Cochran's Q statistic, horizontal pleiotropy was assessed via the MR‐Egger intercept, and leave‐one‐out analyses were conducted to exclude undue influence from individual variants [21].

## 3 Results

### 3.1 Descriptive Analysis

Marked heterogeneity in the delirium reporting proportion was observed across included drugs. Methadone exhibited the highest delirium reporting rate at 6.70% (81/1,218), substantially exceeding the reporting proportion associated with other drugs (Table 1). Elevated reporting proportions were also noted for oxycodone (2.20%), hydromorphone (1.80%), and morphine (1.80%). In contrast, buprenorphine (0.40%) and hydrocodone (0.50%) demonstrated the lowest delirium occurrence.

**TABLE 1:** Features of opioid‐associated delirium.

Drug	Total exposed cases	Delirium cases	Delirium proportion	Mean age	Age SD ()	Male cases	Male percent	Female cases	Female percent
FENTANYL	11,950	92	0.80%	60.9	20.1	51	55.40%	41	44.60%
DEXMEDETOMIDINE	648	7	1.10%	31.5	30.5	6	85.70%	1	14.30%
MORPHINE	6171	112	1.80%	61	17.4	70	62.50%	42	37.50%
METHADONE	1218	81	6.70%	62.6	12.3	55	67.90%	26	32.10%
TRAMADOL	6508	78	1.20%	66.5	23.8	41	52.60%	37	47.40%
OXYCODONE	7678	167	2.20%	66.9	14.1	105	62.90%	62	37.10%
HYDROMORPHONE	1627	29	1.80%	59.8	19.5	16	55.20%	13	44.80%
CODEINE	1600	14	0.90%	76.4	15.3	7	50%	7	50%
HYDROCODONE	2436	12	0.50%	67.6	22	10	83.30%	2	16.70%
BUPRENORPHINE	5872	24	0.40%	72.2	18.3	10	41.70%	14	58.30%

Among adverse event reports involving delirium, age distributions varied by drug exposure. The three highest mean ages were observed for codeine (76.4 ± 15.3 years), followed by buprenorphine (72.2 ± 18.3 years) and oxycodone (66.9 ± 14.1 years). Sex‐specific patterns were also evident. Delirium associated with dexmedetomidine and hydrocodone was predominantly reported in male patients, accounting for 85.70% and 83.30% of cases, respectively. Conversely, a higher proportion of female patients was observed in buprenorphine‐related adverse event reports (58.30%), with tramadol also showing a relatively increased female representation (47.40%).

### 3.2 Disproportionality Analysis

Signal detection using ROR, PRR, and EBGM revealed substantial variability in drug‐delirium association strength. Methadone demonstrated the most pronounced signal despite a moderate number of reports (*n* = 81), with markedly elevated metrics (ROR 28.03, 95% CI 22.35–35.16; PRR 26.24, 95% CI 21.23–32.42; EBGM 25.98, EB05 21.49), indicating a robust association with delirium (Table 2).

**TABLE 2:** Disproportionality analysis of opioid‐associated delirium.

Drug	Delirium cases	ROR	ROR (95% CI)	PRR	PRR (95% CI)	EBGM	EBGM 05	Signal detection
FENTANYL	92	3.05	2.48–3.74	3.03	2.47–3.72	3.01	2.53	Positive
DEXMEDETOMIDINE	7	4.26	2.02–8.97	4.22	2.02–8.82	4.22	2.26	Positive
MORPHINE	112	7.29	6.04–8.8	7.18	5.97–8.63	7.09	6.05	Positive
METHADONE	81	28.03	22.35–35.16	26.24	21.23–32.42	25.98	21.49	Positive
TRAMADOL	78	4.76	3.81–5.96	4.72	3.78–5.89	4.68	3.88	Positive
OXYCODONE	167	8.83	7.56–10.31	8.66	7.44–10.08	8.5	7.46	Positive
HYDROMORPHONE	29	7.09	4.91–10.25	6.98	4.87–10.02	6.96	5.11	Positive
CODEINE	14	3.44	2.03–5.83	3.42	2.03–5.77	3.42	2.2	Positive
HYDROCODONE	12	1.93	1.09–3.41	1.93	1.09–3.39	1.92	1.19	Positive
BUPRENORPHINE	24	1.6	1.07–2.39	1.6	1.07–2.38	1.6	1.14	Positive

**FIGURE 2:**
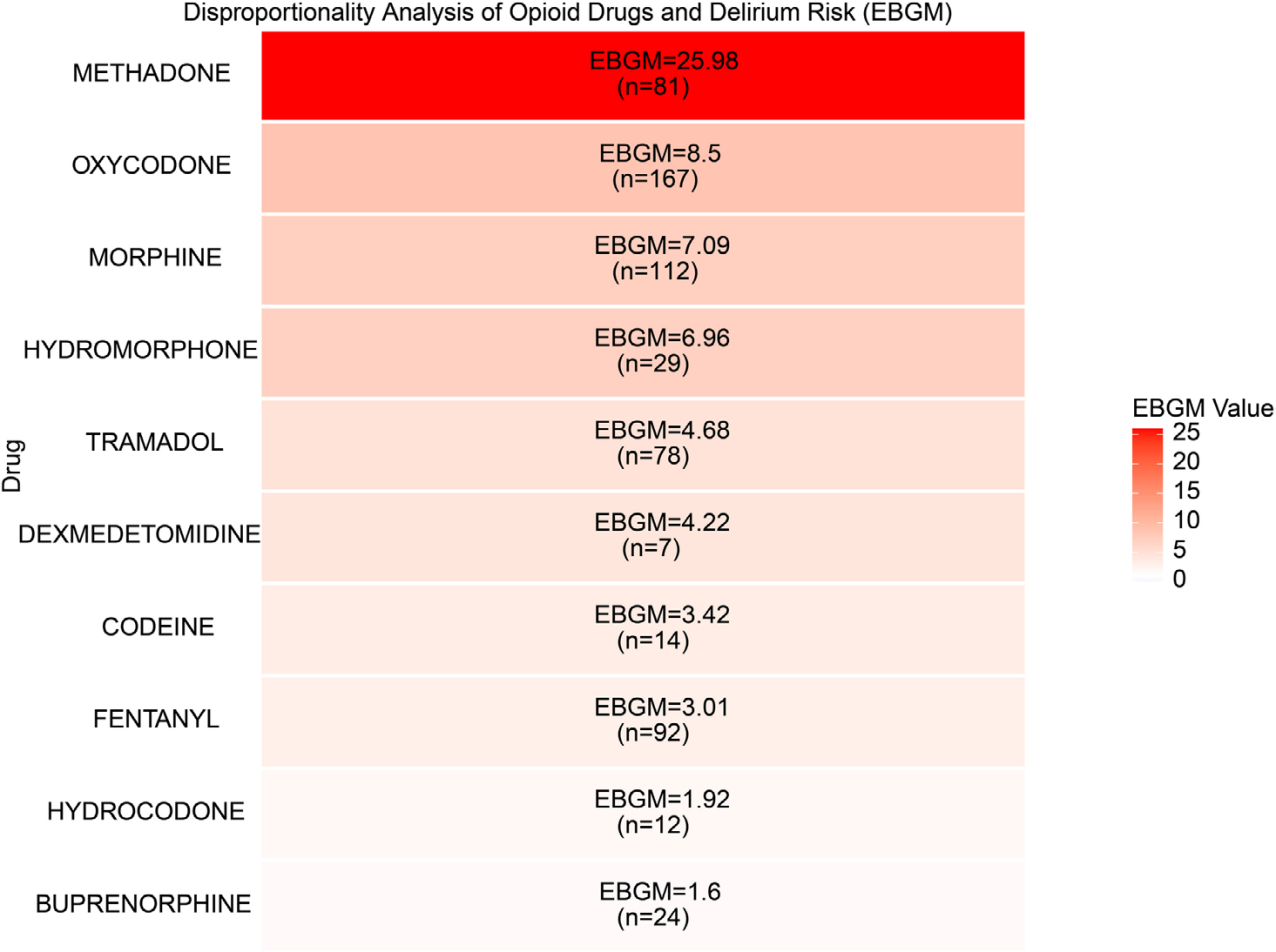
Heat map of disproportionality analysis for opioids using the empirical Bayes geometric mean.

Strong signals were also identified for oxycodone (*n* = 167; ROR 8.83, 95% CI 7.56–10.31), followed by morphine (*n* = 112; ROR 7.29, 95% CI 6.04–8.80) and hydromorphone (*n* = 29; ROR 7.09, 95% CI 4.91–10.25). In comparison, weaker associations were observed for hydrocodone (*n* = 12; ROR 1.93, 95% CI 1.09–3.41) and buprenorphine (*n* = 24; ROR 1.60, 95% CI 1.07–2.39). EBGM‐based heatmap visualization further corroborated strong signals linking methadone, oxycodone, morphine, and hydromorphone with delirium (Figure 2).

### 3.3 Subgroup Analysis

**FIGURE 3:**
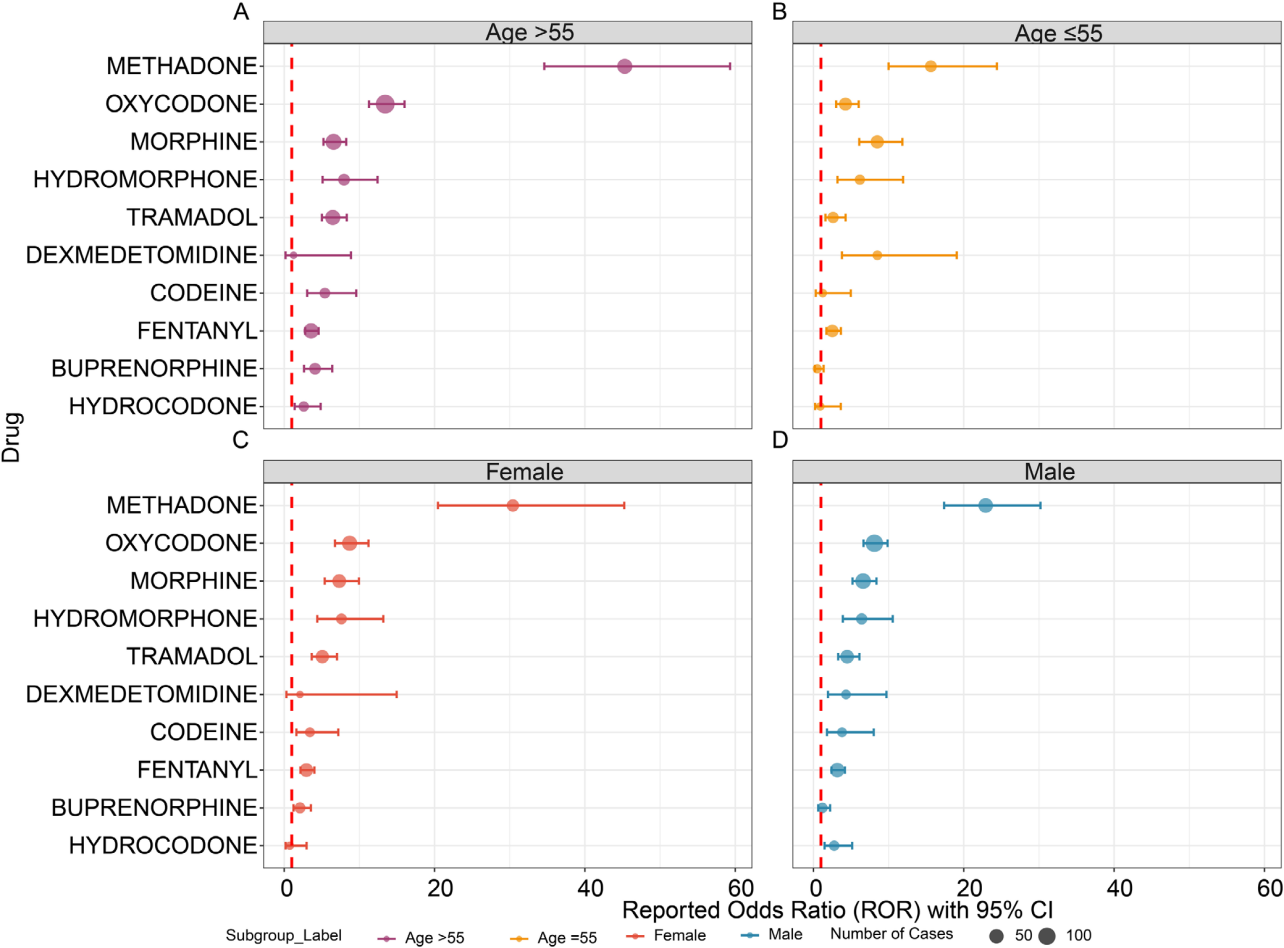
Subgroup analysis of the association between opioid use and delirium risk. (A) Age > 55, (B) Age ≤ 55, (C) Female, and (D) Male.

Stratified disproportionality analyses by age and sex revealed notable effect modification. Among patients aged ≤ 55 years, methadone displayed the strongest delirium signal (ROR 15.623, 95% CI 10.000–24.408), followed by dexmedetomidine (ROR 8.504, 95% CI 3.793–19.065) and morphine (ROR 8.493, 95% CI 6.096–11.831) (Figure 3, Table S4).

In patients older than 55 years, the methadone‐associated risk intensified markedly, with ROR increasing to 45.302 (95% CI 34.601–59.313). Enhanced signals were also observed for other opioids in this age group, led by oxycodone (ROR 13.437, 95% CI 11.281–16.005), hydromorphone (ROR 7.959, 95% CI 5.103–12.415), morphine (ROR 6.565, 95% CI 5.223–8.252), and tramadol (ROR 6.462, 95% CI 5.018–8.321).

Sex‐stratified analyses demonstrated significant delirium signals for methadone and oxycodone in both males and females. Signal strength was consistently higher among female patients. For methadone, ROR values reached 30.420 in females compared with 22.913 in males. A similar pattern was observed for oxycodone, with ROR values of 8.705 in females and 8.107 in males.

### 3.4 Multivariable Logistic Regression Analysis

To further quantify independent risk while accounting for confounding, multivariable logistic regression models were applied. Using non‐target drugs as the reference category, all included opioids were independently associated with an increased risk of delirium. Methadone demonstrated the highest adjusted risk, with an OR of 29.787 (95% CI 23.539–37.156, *p* < 0.001) (Table 3). Elevated risks were also observed for oxycodone (OR 9.257, 95% CI 7.895–10.775, *p* < 0.001), hydromorphone (OR 7.526, 95% CI 5.088–10.661, *p* < 0.001), and morphine (OR 7.063, 95% CI 5.816–8.484, *p* < 0.001). Age and sex emerged as significant covariates. Each additional year of age was associated with a 1.4% increase in delirium risk (OR 1.014, 95% CI 1.013–1.016), while sex also showed a significant association with delirium occurrence (OR 1.735, 95% CI 1.659–1.814).

**TABLE 3:** Multivariable logistic regression analysis of opioid use and delirium risk.

Variable	Regression coefficient	Std Error	OR	95% CI	*p*
Intercept	−7.115	0.039	0.001	0.001–0.001	< 0.001
METHADONE	3.394	0.116	29.787	23.539–37.156	< 0.001
OXYCODONE	2.225	0.079	9.257	7.895–10.775	< 0.001
MORPHINE	1.955	0.096	7.063	5.816–8.484	< 0.001
HYDROMORPHONE	2.018	0.188	7.526	5.088–10.661	< 0.001
TRAMADOL	1.641	0.115	5.161	4.089–6.412	< 0.001
DEXMEDETOMIDINE	1.538	0.381	4.653	1.991–9.059	< 0.001
CODEINE	1.393	0.269	4.026	2.262–6.542	< 0.001
FENTANYL	1.201	0.105	3.323	2.684–4.059	< 0.001
HYDROCODONE	0.731	0.29	2.078	1.111–3.493	0.012
BUPRENORPHINE	0.702	0.205	2.019	1.313–2.944	< 0.001
AGE	0.014	0.001	1.014	1.013–1.016	< 0.001
SEX	0.551	0.023	1.735	1.659–1.814	< 0.001

### 3.5 Identification of Candidate Drug Targets in the FAERS Database

Target prediction was performed for ten drugs identified as high risk in pharmacovigilance analyses, including methadone, oxycodone, morphine, hydromorphone, tramadol, dexmedetomidine, codeine, fentanyl, hydrocodone, and buprenorphine. Predicted drug targets were intersected with delirium‐associated genes, yielding a total of 125 shared candidate genes implicated in delirium pathogenesis (Figure S1, Table S5).

Methadone was linked to multiple targets involved in opioid receptor signaling and neurotransmission, with representative genes including *OPRM1*, *ACHE*, and *NTRK2*. Hydrocodone was associated with a diverse set of targets related to neuronal regulation and neurotransmitter metabolism, exemplified by *WBP2NL*, *MACROD2*, and *COMT*. Morphine showed interactions with several genes implicated in opioid signaling and pharmacokinetic processes, such as *OPRD1*, *CYP2C8*, and *ABCB1*. These representative associations highlight the extensive and diverse molecular‐target landscapes that distinguish individual opioids.

### 3.6 Molecular Mechanisms and Pathway Enrichment Analysis

**FIGURE 4:**
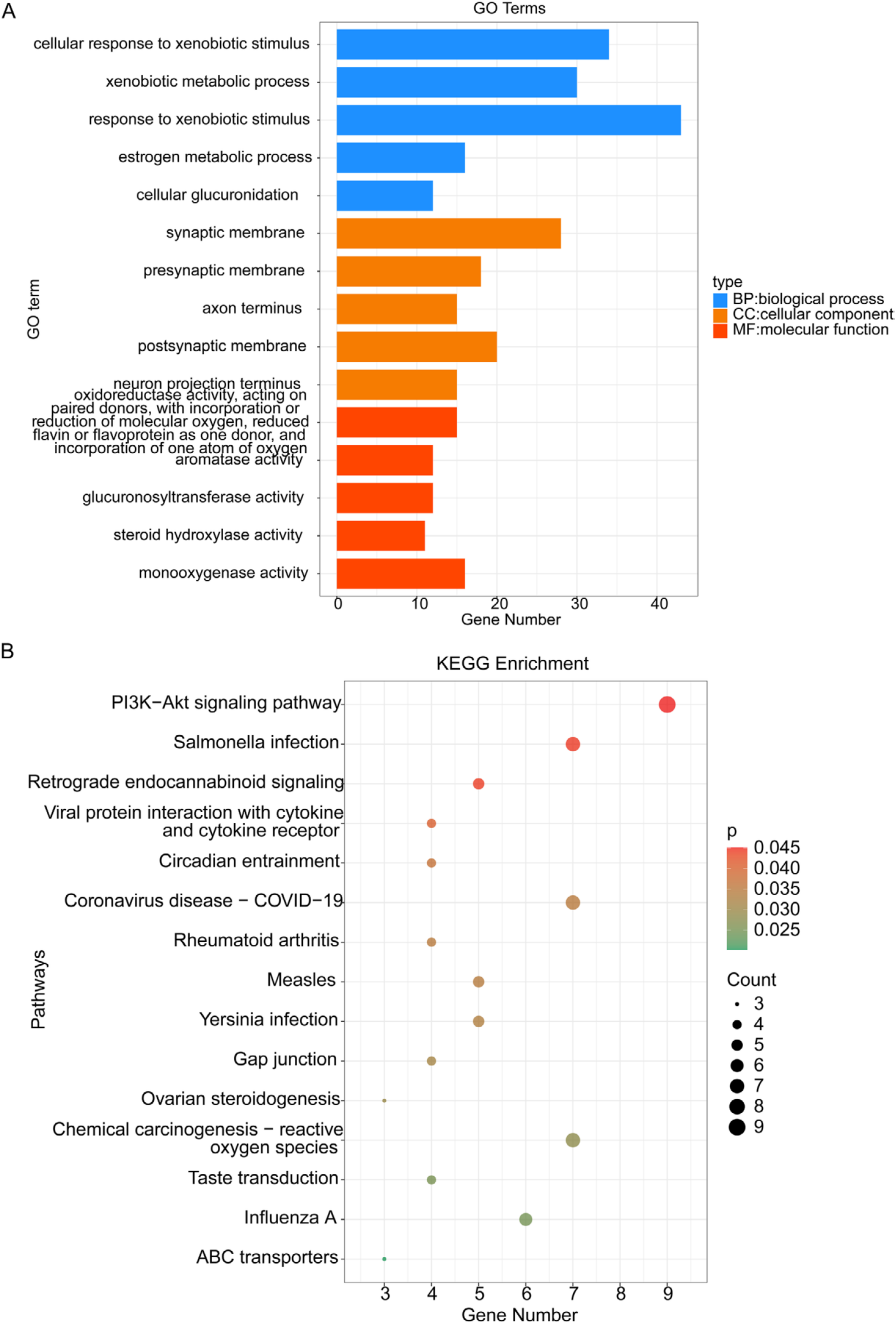
Enrichment analysis. (A) GO enrichment analysis. (B) KEGG enrichment analysis.

To elucidate the biological functions of shared target genes, GO and KEGG enrichment analyses were conducted (Figure 4). GO enrichment analysis yielded a total of 1372 significant terms, comprising 1142 biological processes, 72 cellular components, and 158 molecular functions. For visualization, the top five enriched terms ranked by *p*‐value were selected from each category. Within the biological process domain, significant enrichment was observed in pathways related to xenobiotic handling and metabolic regulation, including cellular response to xenobiotic stimulus, xenobiotic metabolic process, estrogen metabolic process, and cellular glucuronidation. These findings underscore the central role of drug metabolism and detoxification in opioid‐associated delirium.

Cellular component enrichment was predominantly localized to synaptic and neuronal structures, with target genes concentrated in synaptic membranes, presynaptic and postsynaptic membranes, axon termini, and neuron projection termini. This distribution suggested that delirium‐related targets were primarily involved in synaptic transmission and neuronal signaling.

Within the molecular function category, enriched terms were mainly associated with enzymatic activities involved in drug and steroid metabolism, including oxidoreductase activity, aromatase activity, glucuronosyltransferase activity, steroid hydroxylase activity, and monooxygenase activity.

KEGG pathway analysis identified 73 significantly enriched pathways. Visualization of the top 15 pathways revealed prominent enrichment in the PI3K‐Akt signaling pathway and retrograde endocannabinoid signaling, both of which are critically involved in synaptic plasticity, neuroinflammation, and cognitive regulation. Additional enrichment was observed in immune‐ and inflammation‐related pathways, including viral protein interaction with cytokine and cytokine receptor and Salmonella infection pathways, suggesting an interplay between neuroimmune activation and delirium development.

**FIGURE 5:**
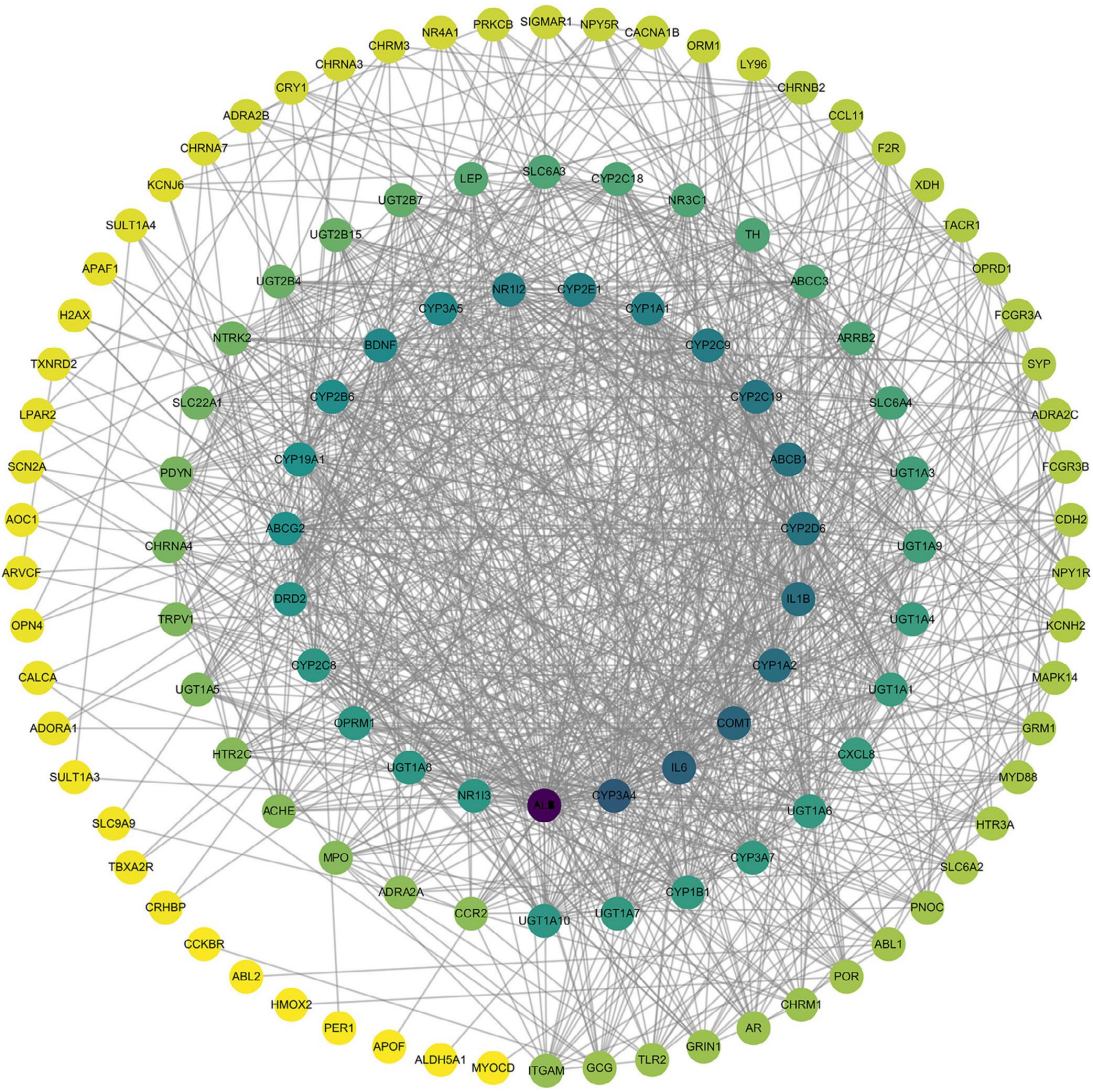
PPI network and functional enrichment of opioid drug targets. Each node represents a protein; edges indicate experimentally validated protein–protein interactions. Node color saturation increases with the number of connections (degree), highlighting hub proteins with the darkest shade.

PPI network analysis demonstrated a highly interconnected structure comprising 117 nodes and 1077 edges, with a clustering coefficient of 0.575, network density of 0.159, and heterogeneity of 0.707 (Figure 5). Hub gene analysis identified *ALB*, *CYP3A4*, *IL6*, *COMT*, *CYP1A2*, and *IL1B* as key regulatory nodes, indicating potential central roles in integrating drug metabolism, inflammatory signaling, and neurotransmitter regulation in opioid‐associated delirium.

### 3.7 MR Analysis

Under stringent instrument selection criteria, independent single‐nucleotide polymorphisms were identified for three candidate exposure genes, *CYP1B1*, *COMT*, and *MYD88*. All selected variants reached genome‐wide significance (*p* < 5 × 10^−8^) and remained mutually independent following linkage disequilibrium pruning, thereby satisfying the relevance and independence assumptions required for MR analyses. For *CYP1B1*, fourteen significantly associated SNPs were retained, distributed across chromosomes 1, 2, 3, 8, 9, 10, 16, and 17. Effect allele frequencies ranged from 0.014 to 0.575, indicating robust genetic instruments with strong exposure‐variant associations (Table S6). Four independent SNPs were selected for *COMT*, primarily located on chromosomes 8 and 22, with effect allele frequencies between 0.040 and 0.582, supporting adequate explanation of genetically determined variation in gene expression. Five genome‐wide significant SNPs were identified for *MYD88*, distributed across chromosomes 3, 5, and 7, with effect allele frequencies ranging from 0.016 to 0.750, demonstrating stable and statistically significant genetic associations with the exposure.

**FIGURE 6:**
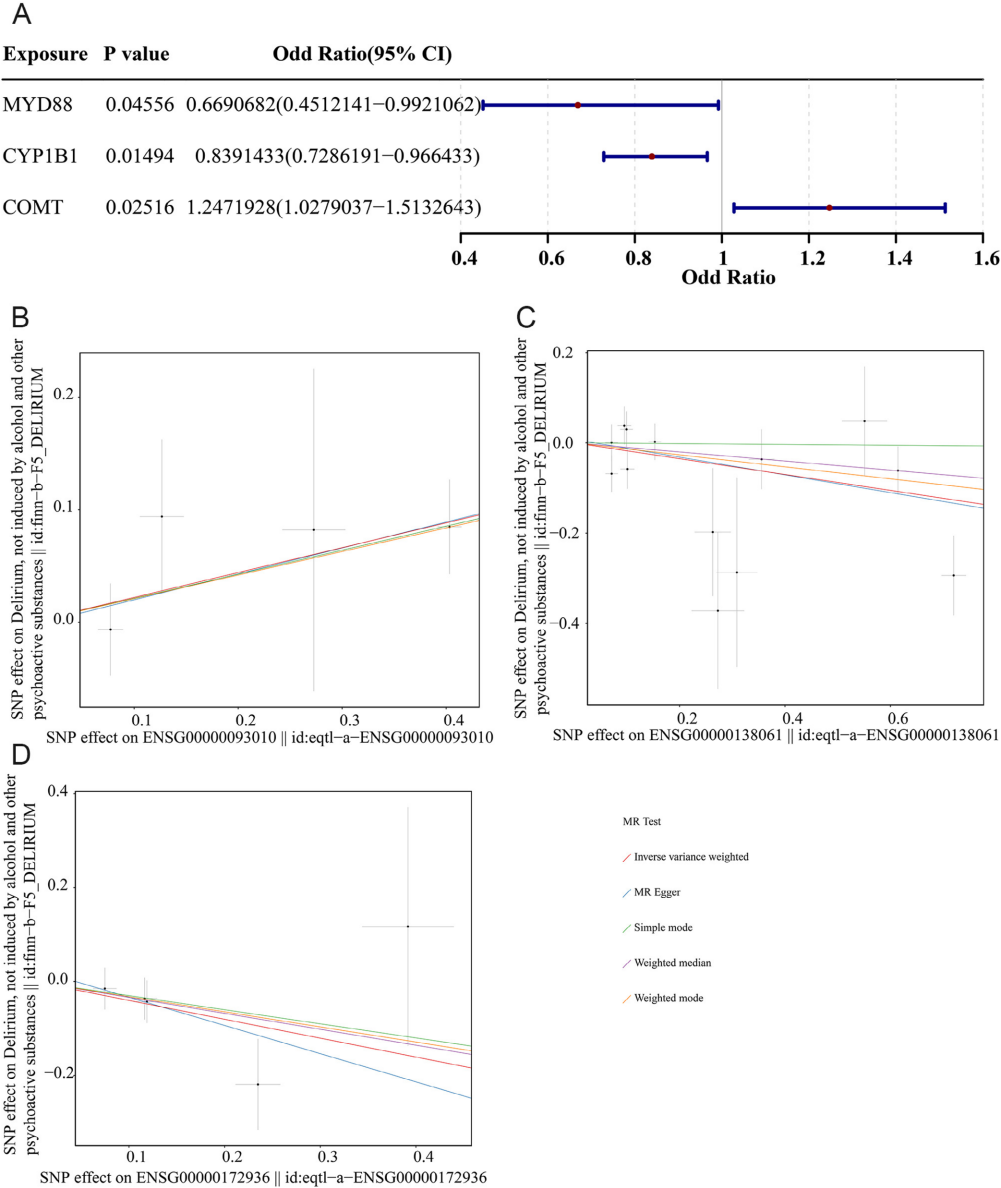
Genes associated with delirium. (A) Forest plot of MR estimates. (B–D) Association analysis between candidate exposure factors and delirium. (B) COMT, (C) CYP1B1, and (D) MYD88. The x‐axis represents the per‐allele effect of each SNP on the exposure, and the y‐axis shows the corresponding effect on the outcome. Colored lines depict the causal estimates derived from different MR algorithms; the slope of each line indicates the magnitude and direction of the causal effect estimated by that specific method.

MR analyses were conducted to assess potential causal relationships between drug target genes and delirium. Statistically significant associations with delirium were identified for three genes, *MYD88* (OR 0.669, 95% CI 0.451–0.992, *p* = 0.046), *CYP1B1* (OR 0.839, 95% CI 0.729–0.966, *p* = 0.015), and *COMT* (OR 1.247, 95% CI 1.028–1.513, *p* = 0.025) (Figure 6A, Table S7). Results obtained using MR‐Egger regression, the weighted median method, the simple mode, and the weighted mode were directionally consistent with the primary inverse variance weighted estimates, further supporting *MYD88* and *CYP1B1* as protective factors against delirium, while indicating *COMT* as a risk‐associated factor for delirium.

Scatter plots generated using five MR methods demonstrated consistent directional effects. Negative regression slopes were observed for *MYD88* and *CYP1B1*, indicating that increased genetically predicted expression of these genes was associated with a reduced risk of delirium, consistent with protective effects (Figure 6B–D). In contrast, a positive regression slope was observed for *COMT*, suggesting a deleterious association and supporting its role as a risk factor for delirium.

For *MYD88* and *CYP1B1*, the majority of SNP‐specific estimates were positioned to the left of the null line in scatter plots, with pooled inverse variance weighted estimates also located on the left side, indicating that genetically instrumented increases in exposure were associated with lower delirium risk (Figure S2). Conversely, most *COMT*‐related SNPs were distributed to the right of the null line, consistent with an increased risk of delirium. Funnel plots showed symmetrical distributions of SNP effects around the pooled estimates, indicating adequate randomization and independent contributions of individual variants (Figure S3). Tests for heterogeneity and horizontal pleiotropy yielded non‐significant results (all *p* > 0.05), suggesting the absence of substantial heterogeneity or directional pleiotropy (Table S8,S9). Leave‐one‐out analyses further demonstrated result stability, as sequential removal of individual SNPs did not materially alter effect estimates, with all remaining estimates consistently positioned on the same side of the null (Figure S4).

## 4 Discussion

In this integrated pharmacovigilance and genetic analysis, we demonstrate substantial heterogeneity in delirium risk across commonly used opioid agents, with methadone conferring a disproportionately high risk compared with other opioids. By combining large‐scale real‐world adverse event data with molecular network analysis and Mendelian randomization, our findings extend beyond signal detection to provide causal prioritization of biological pathways underlying opioid‐associated delirium. Collectively, these results offer a translational framework for individualized risk stratification and prevention.

A key clinical insight from this study is the marked variability in delirium risk among opioid agents. Methadone consistently exhibited the strongest association across disproportionality analyses and multivariable regression models, with risk signals further amplified in older adults and female patients. These findings align with methadone's complex pharmacokinetic profile, including long and variable half‐life, extensive hepatic metabolism, and potential for drug–drug interactions, all of which may predispose susceptible patients to neurotoxicity [22]. Importantly, such agent‐specific risk differentiation is rarely captured in conventional clinical trials, underscoring the value of pharmacovigilance data in informing real‐world prescribing decisions.

Beyond demographic and drug‐level factors, our analyses highlight the biological underpinnings of opioid‐induced delirium. Network pharmacology and pathway enrichment analyses converged on mechanisms related to xenobiotic metabolism, synaptic signaling, and neuroinflammation, with prominent involvement of the PI3K–Akt pathway. These pathways are central to neuronal survival, synaptic plasticity, and inflammatory regulation [23, 24, 25], processes that are critically disrupted during delirium. The enrichment of metabolic and immune‐related pathways suggests that delirium susceptibility may reflect a failure to adequately buffer pharmacological and inflammatory stress within the central nervous system.

Opioid‐associated neuroinflammation may represent an important biological link between opioid exposure and delirium development. Previous studies have shown that opioids can activate microglia and promote the release of inflammatory cytokines, thereby disrupting synaptic function and cognitive regulation [26]. Consistent with this concept, our enrichment and PPI analyses identified several inflammation‐related mediators, including IL6, IL1B, and MYD88. In addition, the PI3K‐Akt pathway identified in KEGG analysis has been implicated not only in neuronal survival and synaptic plasticity but also in inflammatory signaling and oxidative stress regulation [27, 28], suggesting that dysregulated neuroimmune responses may contribute to opioid‐associated delirium.

Genetic causal inference further refined these mechanistic insights. Mendelian randomization analyses identified genetically proxied COMT‐related pathways as a risk factor for delirium, whereas MYD88 and CYP1B1 showed protective associations. COMT plays a pivotal role in catecholamine metabolism [29], and increased COMT‐related activity may disrupt dopaminergic and noradrenergic balance, thereby lowering the threshold for acute cognitive dysfunction. In contrast, MYD88 is a central adaptor in innate immune signaling [30], and its protective association supports a role for restrained neuroinflammatory responses in mitigating delirium risk [31]. CYP1B1, involved in xenobiotic and steroid metabolism [32], may enhance the clearance or detoxification of neuroactive compounds, further buffering against opioid‐induced neurotoxicity. Together, these findings support a model in which delirium emerges from the convergence of impaired neurotransmitter regulation, heightened neuroimmune sensitivity, and limited metabolic reserve.

From a translational perspective, our study has several implications. First, it supports more cautious use of high‐risk opioids, particularly methadone, in older and female patients, with consideration of alternative analgesic strategies or enhanced neurocognitive monitoring. Second, the identification of genetically informed risk pathways raises the possibility of incorporating molecular or genetic information into future risk stratification frameworks. While clinical implementation of genetic screening is premature, these results provide a rationale for prioritizing mechanistic and interventional studies targeting metabolic and inflammatory pathways to prevent opioid‐associated delirium.

This study has limitations. FAERS is a spontaneous reporting system and is inherently subject to reporting bias, under‐reporting, and incomplete clinical information, precluding estimation of true incidence or definitive causal inference at the drug–event level. Although multivariable regression was used to adjust for key covariates, residual confounding cannot be excluded. In addition, Mendelian randomization evaluates the effects of genetically proxied biological pathways rather than drug exposure per se, and the FinnGen delirium phenotype is based on diagnostic coding, which may not capture all clinical presentations of delirium. Nevertheless, the convergence of pharmacovigilance signals, mechanistic enrichment, and genetic causal evidence strengthens the robustness of our conclusions.

## 5 Conclusion

Our analysis of adverse event reports isolates methadone as a significant trigger for delirium, with risk stratification pointing to older adults (> 55) and female patients as the primary at‐risk populations. Consequently, analgesic planning for these individuals requires careful agent selection and strict monitoring for psychiatric side effects. At the molecular level, the disorder seems to arise from a convergence of metabolic and neuroinflammatory pathways, including the PI3K‐Akt axis. This suggests that a patient's risk is heavily influenced by their capacity for drug metabolism and the sensitivity of their neuroimmune system. Causal genetic inquiry further identifies overexpression of *COMT* as a liability, likely due to impaired neurotransmitter clearance, while revealing that *MYD88* and *CYP1B1* exert protective effects. By aiding in xenobiotic clearance and inflammation control, these genes represent promising targets for future interventions aimed at mitigating opioid‐associated neurotoxicity.

## Author Contributions

X.C., Z.J., B.Q., and F.L. conducted the study concept and design. Y.L., M.Z., F.L., and J.J. performed the statistical analysis. F.L., X.C., and J.J. were responsible for data interpretation. X.C. drafted the original version of the manuscript. All authors read and approved the final manuscript.

## Funding

The study was funded through the Yancheng Basic Research Program (Grant No. YCBK2025104).

## Disclosure

The authors have nothing to report.

## Ethics Statement

This study analyzed publicly available, de‐identified summary‐level data and did not involve direct interaction with human participants. Therefore, additional ethical approval or informed consent was not required.

## Conflicts of Interest

The authors declare no conflicts of interest.

## Supporting information


**Figure S1:** Drug‐target interaction network.
**Figure S2:** Causal effect of SNPs for each candidate exposure on delirium.
**Figure S3:** Randomness assessment of candidate exposure factors.
**Figure S4:** Leave‐one‐out sensitivity analyses.

**Table S1:** MedDRA Preferred Terms used for delirium identification.
**Table S2:** Fourfold for signal detection.
**Table S3:** Criteria for signal detection algorithms.
**Table S4:** Subgroup disproportionality analysis stratified by age and sex.
**Table S5:** Shared candidate genes identified from drug‐target and delirium‐related gene intersection analyses.
**Table S6:** Instrumental SNPs used in Mendelian randomization analyses.
**Table S7:** Detailed Mendelian randomization results.
**Table S8:** Heterogeneity test results.
**Table S9:** Horizontal pleiotropy test results.

## Data Availability

The data that support the findings of this study are available from the corresponding author upon reasonable request.
