# Supplementary material for: Integrating Pharmacovigilance Data Mining and Mendelian Randomization to Identify Risk Profiles and Causal Targets of Opioid‐Induced Delirium
Source: CNS Neurosci Ther. 2026 Jun 17;32(6):e70983. doi: 10.1002/cns.70983 (PMC13274230; doi:10.1002/cns.70983)
Supplement: Supplementary file 1 — Figure S1: Drug‐target interaction network. Figure S2: Causal effect of SNPs for each candidate exposure on delirium. Figure S3: Randomness assessment of candidate exposure factors. Figure S4: Leave‐one‐out sensitivity analyses. [file CNS-32-e70983-s002.docx]

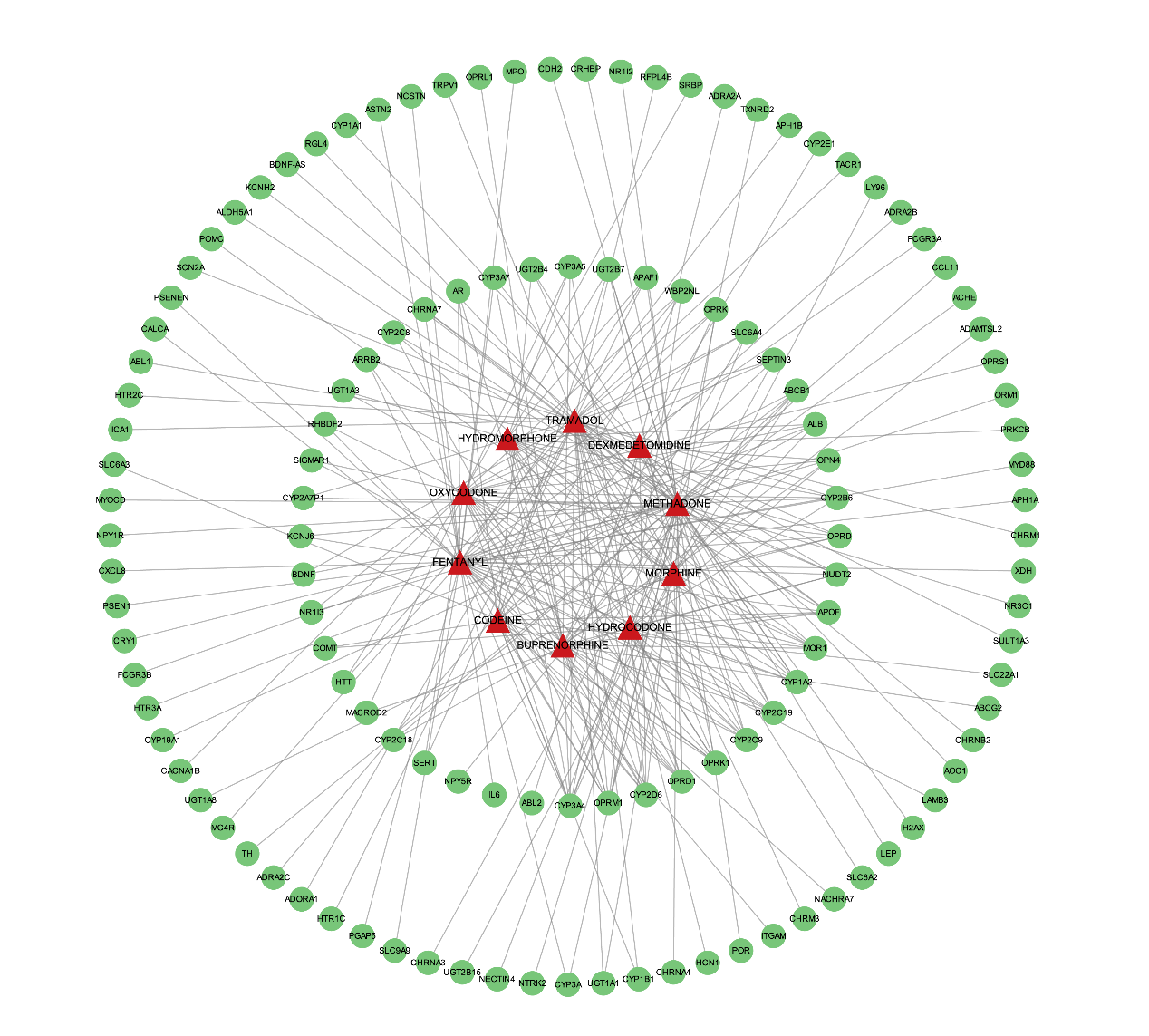


Figure S1. Drug-target interaction network.


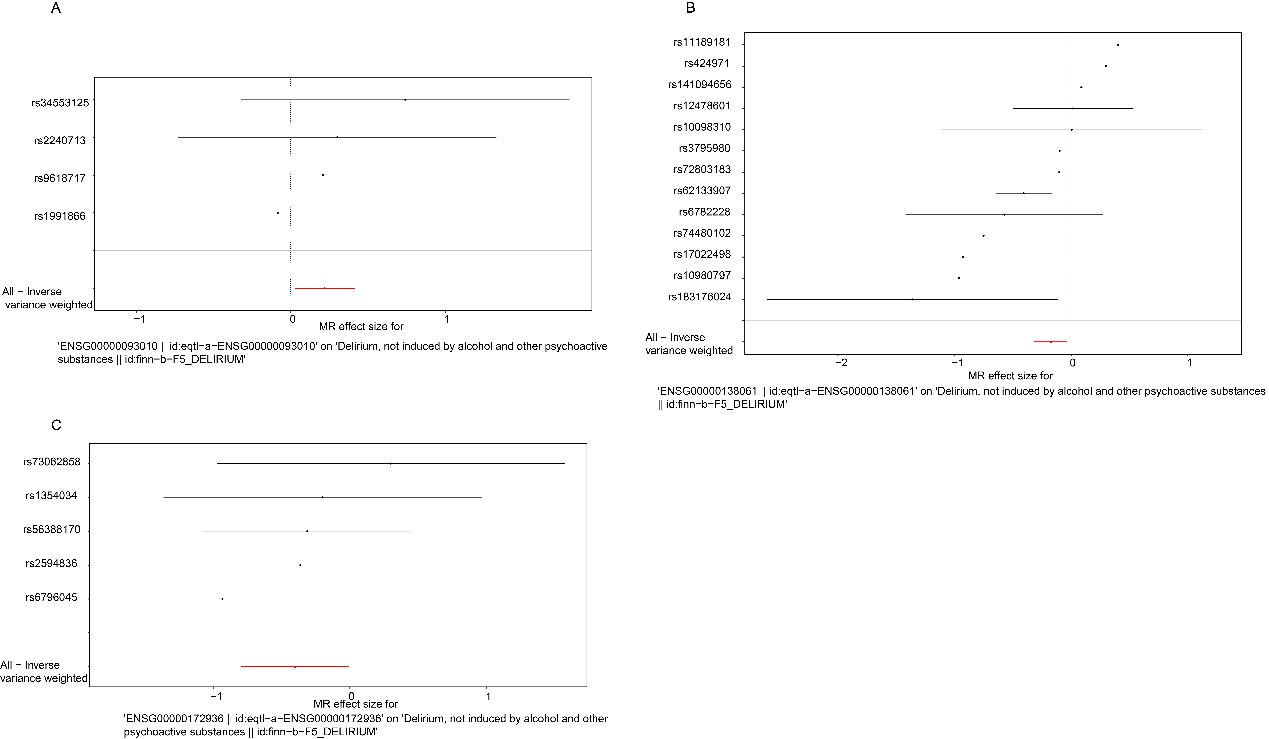


Figure S2. Causal effect of SNPs for each candidate exposure on delirium. (A) COMT. (B) CYP1B1. (C) MYD88. Red dots present the overall estimate obtained by inverse-variance-weighted (IVW) meta-analysis of all SNPs; horizontal bars denote 95 % confidence intervals.


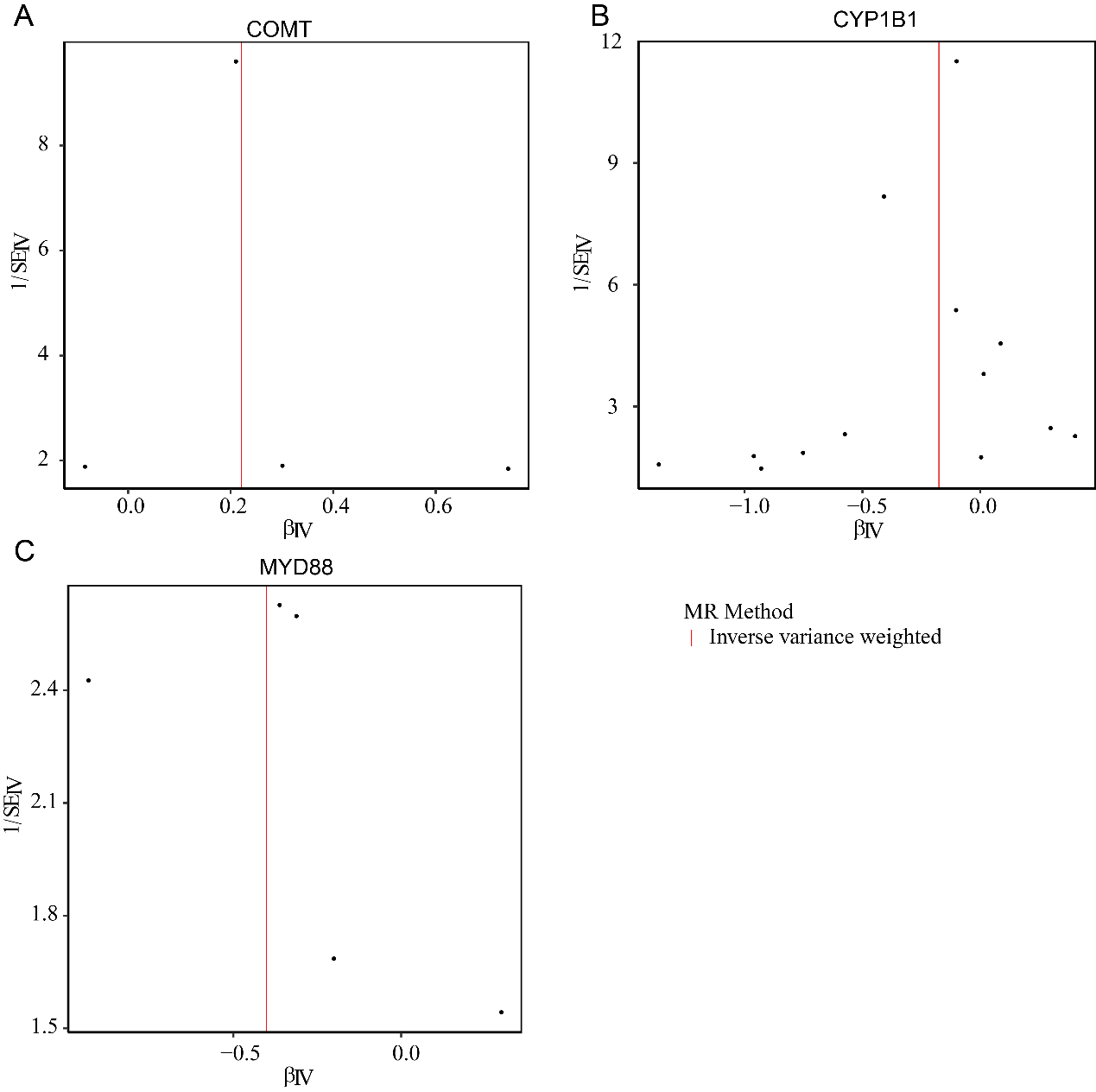


Figure S3. Randomness assessment of candidate exposure factors. (A) COMT. (B) CYP1B1. (C) MYD88. The x-axis shows the β estimate of each instrumental variable, and the y-axis displays the inverse of its standard error.


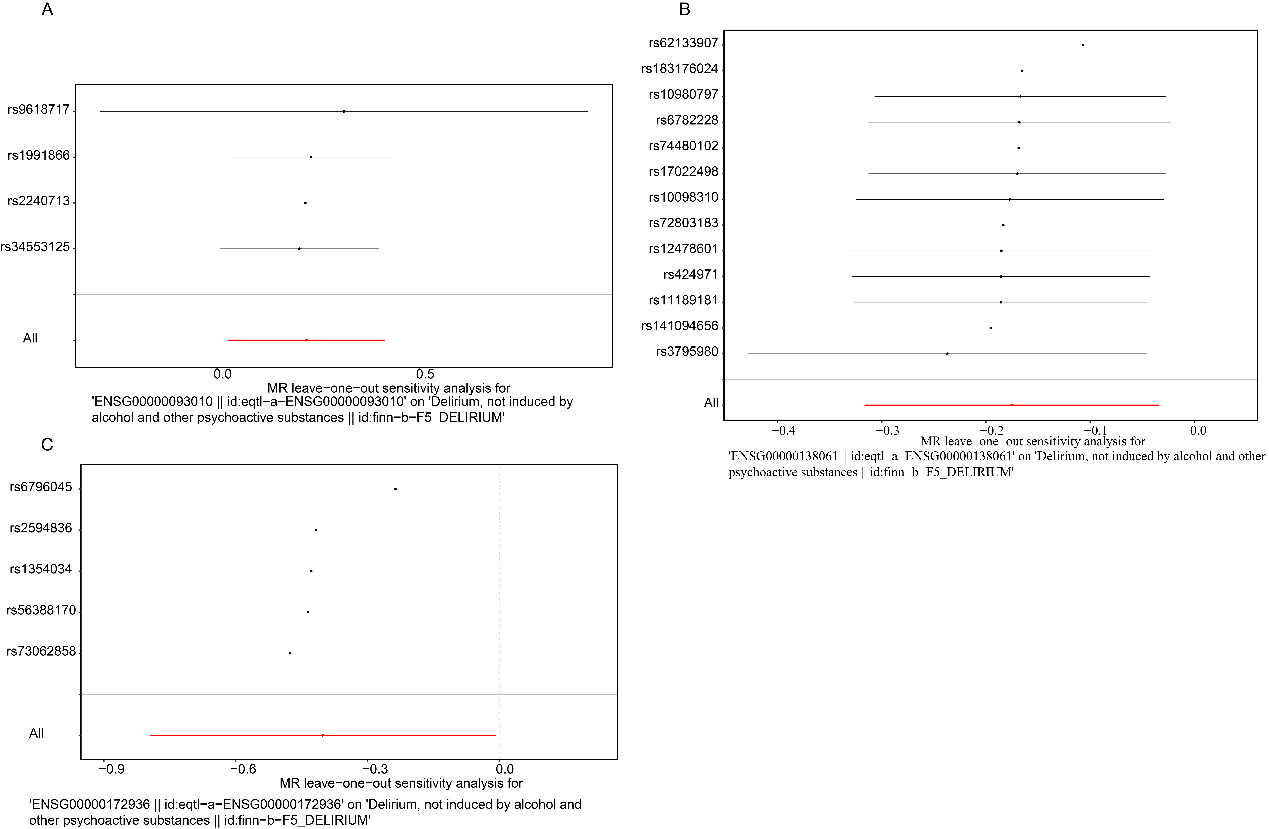


Figure S4. Leave-one-out sensitivity analysis. (A) COMT. (B) CYP1B1. (C) MYD88. Black dots show the IVW estimate after removing each SNP; the red dot indicates the IVW estimate using all SNPs. If the black dots cluster closely around the red dot, the MR estimate is robust. A large horizontal deviation—especially if the effect becomes non-significant or reverses direction-suggests that the overall result is sensitive to the omitted SNP and robustness is questionable.
