# Supplementary material for: Integrating Pharmacovigilance Data Mining and Mendelian Randomization to Identify Risk Profiles and Causal Targets of Opioid‐Induced Delirium
Source: CNS Neurosci Ther. 2026 Jun 17;32(6):e70983. doi: 10.1002/cns.70983 (PMC13274230; doi:10.1002/cns.70983)
Supplement: Supplementary file 2 — Table S1: MedDRA Preferred Terms used for delirium identification. Table S2: Fourfold for signal detection. Table S3: Criteria for signal detection algorithms. Table S4: Subgroup disproportionality analysis stratified by age and sex. Table S5: Shared candidate genes identified from drug‐target and delirium‐related gene intersection analyses. Table S6: Instrumental SNPs used in Mendelian randomization analyses. Table S7: Detailed Mendelian randomization results. Table S8: Heterogeneity test results. Table S9: Horizontal pleiotropy test results. [file CNS-32-e70983-s001.docx]

Table S1. Preferred terms included in the Delirium narrow search.

| SMQ code | Preferred term code | Preferred term name |
| --- | --- | --- |
| 20000133 | 10012218 | Delirium |
| 20000133 | 10012225 | Delirium tremens |
| 20000133 | 10078610 | Postoperative delirium |
| 20000133 | 10072851 | Post-injection delirium-sedation syndrome |
| 20000133 | 10080066 | Intensive care unit delirium |
| 20000133 | 10012294 | Dementia of the Alzheimer type with delirium |
| 20000133 | 10059267 | Febrile delirium |

SMQ: Standardized MedDRA Query.

Table S2. Fourfold for signal detection.

|  | Exposure-related ADEs | Non-exposure related ADEs | Total |
| --- | --- | --- | --- |
| AE | a | b | a + b |
| No AE | c | d | c + d |
| Total | a + c | b + d | N = a+b+c+d |

Note: ADE: adverse drug event. a: number of patients who received opioids and experienced delirium as an ADE. b: number of patients who received opioids but did not experience delirium. c: number of patients who received non-opioid comparators and experienced delirium. d: number of patients who received non-opioid comparators and did not experience delirium. N = a + b + c + d denotes the total number of patients in the study population.

Table S3. Four disproportionality signal detection algorithms.

| Method | Formula | Threshold |
| --- | --- | --- |
| ROR | $ROR=\frac{(a/{c)}}{b/d}=\frac{ad}{bc}$ | a >3 and 95%CI (lower limit)>1 |
|  | $SE\left( lnROR \right)=\sqrt{\frac{1}{a}+\frac{1}{b}+\frac{1}{c}+\frac{1}{d}}$ |  |
|  | $95\%CI=e^{\ln\left( ROR \right)\pm1.96\sqrt{\frac{1}{a}+\frac{1}{b}+\frac{1}{c}+\frac{1}{d}}}$ |  |
| PRR | $PRR=\frac{a/{(a+b)}}{c/{(c+d)}}$ | O=a, a>3, PRR>2 and χ^2^ >4 |
|  | $\chi^{2}=\sum[{{(O-E)}^{2}}/{E]}$ |  |
|  | $E=(a+b）（a+c）/（a+b+c+d）$ |  |
| EBGM | $EBGM=\frac{a/{（a+b+c+d)}}{(a+c)(a+b)}$ | EBGM_05_>2 |
|  | $95\%CI=\text{e}^{\text{ln}(EBGM)\pm1.96\sqrt{\text{（}\frac{1}{\text{a}}+\frac{1}{\text{b}}+\frac{1}{\text{c}}+\frac{1}{\text{d}}\text{）}}}$ |  |

Note: a, number of reports involving both opioid use and delirium; b, number of reports involving opioid use without delirium; c, number of reports involving non-opioid medications (control group) with delirium; and d, number of reports involving neither opioids nor delirium. ROR for Reporting Odds Ratio, PRR for Proportional Reporting Ratio, EBGM for Empirical Bayes Geometric Mean, CI for Confidence Interval, SE for Standard Error, lnROR for natural logarithm, χ^2^ for chi-square statistic, O for Observed number of cases, E for Expected number of cases, IC for Information Component, and EBGM_05_ for the lower 5th percentile of the EBGM posterior distribution.

Table S4. Subgroup analysis of the association between opioid use and delirium risk.

| Drug | Subgroup | Subgroup Type | Cases | ROR | ROR lower | ROR upper |
| --- | --- | --- | --- | --- | --- | --- |
| FENTANYL | ≤55 | Age | 28 | 2.512 | 1.729 | 3.649 |
| DEXMEDETOMIDINE | ≤55 | Age | 6 | 8.504 | 3.793 | 19.065 |
| MORPHINE | ≤55 | Age | 36 | 8.493 | 6.096 | 11.831 |
| METHADONE | ≤55 | Age | 20 | 15.623 | 10 | 24.408 |
| TRAMADOL | ≤55 | Age | 16 | 2.614 | 1.597 | 4.279 |
| OXYCODONE | ≤55 | Age | 33 | 4.263 | 3.02 | 6.018 |
| HYDROMORPHONE | ≤55 | Age | 9 | 6.176 | 3.198 | 11.928 |
| CODEINE | ≤55 | Age | 2 | 1.24 | 0.309 | 4.969 |
| HYDROCODONE | ≤55 | Age | 2 | 0.908 | 0.227 | 3.638 |
| BUPRENORPHINE | ≤55 | Age | 4 | 0.508 | 0.19 | 1.356 |
| FENTANYL | >55 | Age | 64 | 3.575 | 2.79 | 4.58 |
| DEXMEDETOMIDINE | >55 | Age | 1 | 1.246 | 0.175 | 8.884 |
| MORPHINE | >55 | Age | 76 | 6.565 | 5.223 | 8.252 |
| METHADONE | >55 | Age | 61 | 45.302 | 34.601 | 59.313 |
| TRAMADOL | >55 | Age | 62 | 6.462 | 5.018 | 8.321 |
| OXYCODONE | >55 | Age | 134 | 13.437 | 11.281 | 16.005 |
| HYDROMORPHONE | >55 | Age | 20 | 7.959 | 5.103 | 12.415 |
| CODEINE | >55 | Age | 12 | 5.413 | 3.058 | 9.584 |
| HYDROCODONE | >55 | Age | 10 | 2.6 | 1.394 | 4.847 |
| BUPRENORPHINE | >55 | Age | 20 | 4.107 | 2.64 | 6.388 |
| FENTANYL | Male | Gender | 51 | 3.175 | 2.405 | 4.191 |
| DEXMEDETOMIDINE | Male | Gender | 6 | 4.335 | 1.935 | 9.714 |
| MORPHINE | Male | Gender | 70 | 6.604 | 5.201 | 8.386 |
| METHADONE | Male | Gender | 55 | 22.913 | 17.382 | 30.203 |
| TRAMADOL | Male | Gender | 41 | 4.487 | 3.291 | 6.117 |
| OXYCODONE | Male | Gender | 105 | 8.107 | 6.662 | 9.865 |
| HYDROMORPHONE | Male | Gender | 16 | 6.42 | 3.908 | 10.546 |
| CODEINE | Male | Gender | 7 | 3.804 | 1.803 | 8.023 |
| HYDROCODONE | Male | Gender | 10 | 2.762 | 1.481 | 5.153 |
| BUPRENORPHINE | Male | Gender | 10 | 1.193 | 0.641 | 2.221 |
| FENTANYL | Female | Gender | 41 | 2.951 | 2.167 | 4.019 |
| DEXMEDETOMIDINE | Female | Gender | 1 | 2.098 | 0.294 | 14.958 |
| MORPHINE | Female | Gender | 42 | 7.321 | 5.389 | 9.945 |
| METHADONE | Female | Gender | 26 | 30.42 | 20.456 | 45.237 |
| TRAMADOL | Female | Gender | 37 | 5.071 | 3.663 | 7.022 |
| OXYCODONE | Female | Gender | 62 | 8.705 | 6.758 | 11.213 |
| HYDROMORPHONE | Female | Gender | 13 | 7.618 | 4.402 | 13.185 |
| CODEINE | Female | Gender | 7 | 3.42 | 1.625 | 7.197 |
| HYDROCODONE | Female | Gender | 2 | 0.744 | 0.186 | 2.978 |
| BUPRENORPHINE | Female | Gender | 14 | 2.099 | 1.241 | 3.552 |

Table S5. Putative gene targets identified from drug-target interaction.

| drug | gene |
| --- | --- |
| OXYCODONE | WBP2NL |
| OXYCODONE | OPRD1 |
| OXYCODONE | SEPTIN3 |
| OXYCODONE | OPRK1 |
| OXYCODONE | OPRM1 |
| OXYCODONE | OPN4 |
| OXYCODONE | NR1I3 |
| OXYCODONE | CYP1A2 |
| OXYCODONE | CYP2B6 |
| OXYCODONE | MACROD2 |
| OXYCODONE | CYP2C19 |
| OXYCODONE | RHBDF2 |
| OXYCODONE | CYP2C9 |
| TRAMADOL | BDNF |
| TRAMADOL | CYP1A2 |
| TRAMADOL | CYP2C19 |
| TRAMADOL | HTR2C |
| TRAMADOL | OPRK1 |
| TRAMADOL | WBP2NL |
| TRAMADOL | CYP2C9 |
| TRAMADOL | SLC6A4 |
| TRAMADOL | SLC6A2 |
| TRAMADOL | NUDT2 |
| TRAMADOL | OPRD1 |
| TRAMADOL | CYP2D6 |
| TRAMADOL | RGL4 |
| TRAMADOL | APAF1 |
| TRAMADOL | CYP1B1 |
| TRAMADOL | ARRB2 |
| TRAMADOL | HCN1 |
| TRAMADOL | SULT1A3 |
| TRAMADOL | CYP2A7P1 |
| TRAMADOL | RFPL4B |
| TRAMADOL | OPRM1 |
| TRAMADOL | SEPTIN3 |
| TRAMADOL | ICA1 |
| TRAMADOL | APOF |
| BUPRENORPHINE | CYP3A5 |
| BUPRENORPHINE | OPRM1 |
| BUPRENORPHINE | OPN4 |
| BUPRENORPHINE | APOF |
| BUPRENORPHINE | ADAMTSL2 |
| BUPRENORPHINE | OPRK1 |
| BUPRENORPHINE | AR |
| BUPRENORPHINE | NUDT2 |
| BUPRENORPHINE | SLC6A3 |
| BUPRENORPHINE | ARRB2 |
| BUPRENORPHINE | OPRD1 |
| BUPRENORPHINE | SLC6A4 |
| HYDROCODONE | OPRD1 |
| HYDROCODONE | WBP2NL |
| HYDROCODONE | SEPTIN3 |
| HYDROCODONE | NUDT2 |
| HYDROCODONE | CYP3A5 |
| HYDROCODONE | MACROD2 |
| HYDROCODONE | OPRM1 |
| HYDROCODONE | COMT |
| HYDROCODONE | APOF |
| HYDROCODONE | CYP2B6 |
| HYDROCODONE | OPRK1 |
| HYDROCODONE | CYP2C19 |
| HYDROCODONE | OPN4 |
| HYDROCODONE | CYP1A2 |
| HYDROCODONE | CYP3A4 |
| HYDROCODONE | CYP2C9 |
| METHADONE | OPRM1 |
| METHADONE | ACHE |
| METHADONE | NR1I2 |
| METHADONE | BDNF-AS |
| METHADONE | NR1I3 |
| METHADONE | CRY1 |
| METHADONE | BDNF |
| METHADONE | NECTIN4 |
| METHADONE | POR |
| METHADONE | NR3C1 |
| METHADONE | KCNJ6 |
| METHADONE | CRHBP |
| METHADONE | KCNH2 |
| METHADONE | MYOCD |
| METHADONE | TH |
| METHADONE | NTRK2 |
| METHADONE | CYP3A4 |
| METHADONE | LEP |
| METHADONE | NUDT2 |
| METHADONE | APAF1 |
| METHADONE | TXNRD2 |
| METHADONE | APOF |
| METHADONE | ALDH5A1 |
| METHADONE | UGT2B7 |
| METHADONE | ABCB1 |
| METHADONE | CYP2B6 |
| METHADONE | NPY5R |
| METHADONE | H2AX |
| METHADONE | CYP2A7P1 |
| METHADONE | CCL11 |
| METHADONE | CDH2 |
| METHADONE | CYP3A |
| METHADONE | NPY1R |
| METHADONE | SLC6A4 |
| CODEINE | CYP3A4 |
| CODEINE | OPRM1 |
| CODEINE | CYP1B1 |
| CODEINE | CYP2D6 |
| CODEINE | AR |
| CODEINE | WBP2NL |
| CODEINE | APOF |
| CODEINE | SEPTIN3 |
| CODEINE | OPRD1 |
| CODEINE | OPRK1 |
| CODEINE | COMT |
| CODEINE | ABCB1 |
| FENTANYL | CXCL8 |
| FENTANYL | CYP3A |
| FENTANYL | CYP2B6 |
| FENTANYL | OPRD1 |
| FENTANYL | ITGAM |
| FENTANYL | XDH |
| FENTANYL | ARRB2 |
| FENTANYL | CYP3A4 |
| FENTANYL | MPO |
| FENTANYL | CYP2D6 |
| FENTANYL | CALCA |
| FENTANYL | FCGR3B |
| FENTANYL | PGAP6 |
| FENTANYL | AR |
| FENTANYL | CYP1A2 |
| FENTANYL | LAMB3 |
| FENTANYL | MYD88 |
| FENTANYL | APOF |
| FENTANYL | CYP2C19 |
| FENTANYL | ASTN2 |
| FENTANYL | OPRM1 |
| FENTANYL | APAF1 |
| FENTANYL | CYP2C9 |
| FENTANYL | RHBDF2 |
| FENTANYL | SLC9A9 |
| FENTANYL | IL6 |
| FENTANYL | OPN4 |
| FENTANYL | NUDT2 |
| FENTANYL | KCNJ6 |
| FENTANYL | FCGR3A |
| HYDROMORPHONE | NUDT2 |
| HYDROMORPHONE | OPRK1 |
| HYDROMORPHONE | CYP2B6 |
| HYDROMORPHONE | APOF |
| HYDROMORPHONE | OPRM1 |
| HYDROMORPHONE | CYP1A2 |
| HYDROMORPHONE | CYP2D6 |
| HYDROMORPHONE | CYP3A5 |
| HYDROMORPHONE | APAF1 |
| HYDROMORPHONE | OPRD1 |
| HYDROMORPHONE | CYP2C9 |
| HYDROMORPHONE | OPN4 |
| HYDROMORPHONE | CYP3A4 |
| DEXMEDETOMIDINE | ADRA2B |
| DEXMEDETOMIDINE | CYP3A4 |
| DEXMEDETOMIDINE | CYP2C9 |
| DEXMEDETOMIDINE | ABL1 |
| DEXMEDETOMIDINE | CYP1A2 |
| DEXMEDETOMIDINE | ADRA2C |
| DEXMEDETOMIDINE | CYP2C19 |
| DEXMEDETOMIDINE | ABL2 |
| DEXMEDETOMIDINE | AOC1 |
| DEXMEDETOMIDINE | PRKCB |
| DEXMEDETOMIDINE | ADRA2A |
| METHADONE | OPRD1 |
| METHADONE | CHRNA7 |
| METHADONE | HTR3A |
| METHADONE | CHRNA3 |
| METHADONE | CHRNA4 |
| METHADONE | CHRNB2 |
| METHADONE | CYP2C19 |
| METHADONE | CYP3A7 |
| METHADONE | CYP2D6 |
| METHADONE | CYP2C8 |
| METHADONE | CYP19A1 |
| METHADONE | CYP1A2 |
| METHADONE | CYP2C18 |
| METHADONE | CYP2C9 |
| METHADONE | UGT2B4 |
| METHADONE | ORM1 |
| METHADONE | ALB |
| OXYCODONE | CACNA1B |
| OXYCODONE | CYP2D6 |
| OXYCODONE | CYP3A4 |
| OXYCODONE | CYP3A5 |
| OXYCODONE | ALB |
| OXYCODONE | SIGMAR1 |
| OXYCODONE | OPRS1 |
| OXYCODONE | SRBP |
| OXYCODONE | OPRD |
| OXYCODONE | MOR1 |
| OXYCODONE | SLC6A4 |
| OXYCODONE | HTT |
| OXYCODONE | SERT |
| MORPHINE | OPRM1 |
| MORPHINE | OPRK1 |
| MORPHINE | OPRD1 |
| MORPHINE | LY96 |
| MORPHINE | CYP2C8 |
| MORPHINE | CYP3A4 |
| MORPHINE | UGT2B7 |
| MORPHINE | UGT1A1 |
| MORPHINE | UGT1A8 |
| MORPHINE | UGT2B15 |
| MORPHINE | UGT2B4 |
| MORPHINE | UGT1A3 |
| MORPHINE | ALB |
| MORPHINE | ABCB1 |
| MORPHINE | OPRD |
| MORPHINE | OPRK |
| MORPHINE | MOR1 |
| MORPHINE | POMC |
| HYDROMORPHONE | UGT1A3 |
| HYDROMORPHONE | UGT2B7 |
| HYDROMORPHONE | ALB |
| HYDROMORPHONE | OPRD |
| HYDROMORPHONE | MOR1 |
| BUPRENORPHINE | OPRL1 |
| BUPRENORPHINE | CYP3A4 |
| BUPRENORPHINE | CYP2C9 |
| BUPRENORPHINE | CYP2C8 |
| BUPRENORPHINE | CYP3A7 |
| BUPRENORPHINE | CYP2D6 |
| BUPRENORPHINE | CYP2C18 |
| BUPRENORPHINE | CYP2C19 |
| BUPRENORPHINE | ABCB1 |
| BUPRENORPHINE | ABCG2 |
| BUPRENORPHINE | OPRK |
| BUPRENORPHINE | MOR1 |
| TRAMADOL | SCN2A |
| TRAMADOL | ADORA1 |
| TRAMADOL | CHRNA7 |
| TRAMADOL | CHRM3 |
| TRAMADOL | CHRM1 |
| TRAMADOL | TACR1 |
| TRAMADOL | TRPV1 |
| TRAMADOL | CYP2B6 |
| TRAMADOL | UGT1A1 |
| TRAMADOL | CYP3A4 |
| TRAMADOL | HTR1C |
| TRAMADOL | OPRD |
| TRAMADOL | OPRK |
| TRAMADOL | MOR1 |
| TRAMADOL | HTT |
| TRAMADOL | SERT |
| DEXMEDETOMIDINE | CYP2E1 |
| DEXMEDETOMIDINE | CYP1A1 |
| DEXMEDETOMIDINE | CYP2D6 |
| CODEINE | UGT2B7 |
| CODEINE | UGT2B4 |
| CODEINE | SLC22A1 |
| CODEINE | OPRD |
| FENTANYL | OPRK1 |
| FENTANYL | ABCB1 |
| FENTANYL | CYP3A7 |
| FENTANYL | ALB |
| FENTANYL | MC4R |
| FENTANYL | OPRD |
| FENTANYL | OPRK |
| FENTANYL | MOR1 |
| FENTANYL | CHRNA7 |
| FENTANYL | NACHRA7 |
| FENTANYL | APH1A |
| FENTANYL | APH1B |
| FENTANYL | NCSTN |
| FENTANYL | PSENEN |
| FENTANYL | PSEN1 |
| HYDROCODONE | SIGMAR1 |
| HYDROCODONE | CYP2D6 |
| HYDROCODONE | MOR1 |

Table S6. Detailed information on SNPs independently associated with the exposure factor.

| id.exposure | chr | SNP | Effect allele | Other allele | eaf | beta | se.exposure | pval.exposure |
| --- | --- | --- | --- | --- | --- | --- | --- | --- |
| CYP1B1 | 2 | rs3795980 | T | G | 0.143917 | 0.614376 | 0.0161485 | 1.00E-200 |
| CYP1B1 | 2 | rs62133907 | A | G | 0.0652975 | -0.720045 | 0.0233165 | 1.00E-200 |
| CYP1B1 | 2 | rs72803183 | G | A | 0.0622135 | -0.355639 | 0.024458 | 6.66E-48 |
| CYP1B1 | 1 | rs141094656 | C | T | 0.0195137 | 0.551082 | 0.0427775 | 5.65E-38 |
| CYP1B1 | 2 | rs12478601 | T | C | 0.574604 | -0.153089 | 0.0119691 | 1.86E-37 |
| CYP1B1 | 16 | rs424971 | C | T | 0.421674 | -0.0993326 | 0.0120233 | 1.43E-16 |
| CYP1B1 | 2 | rs17022498 | C | G | 0.0238782 | -0.308693 | 0.0388978 | 2.08E-15 |
| CYP1B1 | 17 | rs74480102 | A | G | 0.0309155 | -0.262926 | 0.0343142 | 1.82E-14 |
| CYP1B1 | 3 | rs6782228 | C | G | 0.273721 | -0.100959 | 0.0133216 | 3.49E-14 |
| CYP1B1 | 10 | rs11189181 | G | A | 0.297492 | 0.095192 | 0.0129944 | 2.38E-13 |
| CYP1B1 | 9 | rs10980797 | G | A | 0.472236 | -0.0711707 | 0.0119068 | 2.27E-09 |
| CYP1B1 | 8 | rs10098310 | A | G | 0.575007 | -0.0710502 | 0.0120249 | 3.45E-09 |
| CYP1B1 | 2 | rs2630709 | T | A | 0.493998 | 0.0656033 | 0.0118915 | 3.45E-08 |
| CYP1B1 | 2 | rs183176024 | T | C | 0.0143742 | -0.272699 | 0.0499504 | 4.77E-08 |
| COMT | 22 | rs9618717 | G | A | 0.445755 | 0.403213 | 0.0114831 | 1.00E-200 |
| COMT | 22 | rs2240713 | T | C | 0.0398406 | -0.272727 | 0.0303438 | 2.52E-19 |
| COMT | 8 | rs1991866 | C | G | 0.582429 | -0.077274 | 0.0120511 | 1.43E-10 |
| COMT | 22 | rs34553125 | T | G | 0.0867206 | 0.126665 | 0.0211217 | 2.01E-09 |
| MYD88 | 3 | rs6796045 | T | A | 0.0686759 | 0.234835 | 0.0234506 | 1.32E-23 |
| MYD88 | 7 | rs56388170 | T | G | 0.289189 | -0.116374 | 0.0130907 | 6.12E-19 |
| MYD88 | 5 | rs2594836 | A | G | 0.749651 | 0.118738 | 0.0137022 | 4.49E-18 |
| MYD88 | 3 | rs73062858 | T | C | 0.0155649 | 0.391662 | 0.0479684 | 3.22E-16 |
| MYD88 | 3 | rs1354034 | C | T | 0.618137 | -0.0748289 | 0.0122341 | 9.58E-10 |

Table S7. Mendelian randomization estimates based on five complementary methods.

| SYMBOL | id.exposure | id.outcome | method | nsnp | pval | or | or_lci95 | or_uci95 |
| --- | --- | --- | --- | --- | --- | --- | --- | --- |
| CYP1B1 | eqtl-a-ENSG00000138061 | finn-b-F5_DELIRIUM | MR Egger | 13 | 0.090956306 | 0.821894462 | 0.667864524 | 1.011448404 |
| CYP1B1 | eqtl-a-ENSG00000138061 | finn-b-F5_DELIRIUM | Weighted median | 13 | 0.203099289 | 0.904159458 | 0.774220549 | 1.055906261 |
| CYP1B1 | eqtl-a-ENSG00000138061 | finn-b-F5_DELIRIUM | Inverse variance weighted | 13 | 0.014939526 | 0.839143324 | 0.728619076 | 0.966432998 |
| CYP1B1 | eqtl-a-ENSG00000138061 | finn-b-F5_DELIRIUM | Simple mode | 13 | 0.95133494 | 0.991128581 | 0.748882898 | 1.311734943 |
| CYP1B1 | eqtl-a-ENSG00000138061 | finn-b-F5_DELIRIUM | Weighted mode | 13 | 0.093333104 | 0.875641209 | 0.759120438 | 1.010047273 |
| COMT | eqtl-a-ENSG00000093010 | finn-b-F5_DELIRIUM | MR Egger | 4 | 0.316124759 | 1.258997422 | 0.895630539 | 1.769786132 |
| COMT | eqtl-a-ENSG00000093010 | finn-b-F5_DELIRIUM | Weighted median | 4 | 0.038503802 | 1.237918694 | 1.011352092 | 1.515241533 |
| COMT | eqtl-a-ENSG00000093010 | finn-b-F5_DELIRIUM | Inverse variance weighted | 4 | 0.025159012 | 1.247192835 | 1.02790369 | 1.513264308 |
| COMT | eqtl-a-ENSG00000093010 | finn-b-F5_DELIRIUM | Simple mode | 4 | 0.40387244 | 1.238001142 | 0.803972021 | 1.906343488 |
| COMT | eqtl-a-ENSG00000093010 | finn-b-F5_DELIRIUM | Weighted mode | 4 | 0.132050162 | 1.233179976 | 1.009796891 | 1.505978943 |
| MYD88 | eqtl-a-ENSG00000172936 | finn-b-F5_DELIRIUM | MR Egger | 5 | 0.340659252 | 0.548761889 | 0.193794768 | 1.55390991 |
| MYD88 | eqtl-a-ENSG00000172936 | finn-b-F5_DELIRIUM | Weighted median | 5 | 0.177209299 | 0.712666959 | 0.435716938 | 1.16565171 |
| MYD88 | eqtl-a-ENSG00000172936 | finn-b-F5_DELIRIUM | Inverse variance weighted | 5 | 0.045561835 | 0.669068218 | 0.451214081 | 0.992106186 |
| MYD88 | eqtl-a-ENSG00000172936 | finn-b-F5_DELIRIUM | Simple mode | 5 | 0.457058679 | 0.740786206 | 0.362349198 | 1.514462307 |
| MYD88 | eqtl-a-ENSG00000172936 | finn-b-F5_DELIRIUM | Weighted mode | 5 | 0.379854381 | 0.724630578 | 0.382046929 | 1.374410931 |

Table S8. Heterogeneity test.

| id.exposure | id.outcome | method | Q | Q_df | Q_pval |
| --- | --- | --- | --- | --- | --- |
| eqtl-a-ENSG00000138061 | finn-b-F5_DELIRIUM | MR Egger | 18.19056062 | 11 | 0.077262962 |
| eqtl-a-ENSG00000138061 | finn-b-F5_DELIRIUM | Inverse variance weighted | 18.31826191 | 12 | 0.106363171 |
| eqtl-a-ENSG00000093010 | finn-b-F5_DELIRIUM | MR Egger | 1.280400358 | 2 | 0.527186882 |
| eqtl-a-ENSG00000093010 | finn-b-F5_DELIRIUM | Inverse variance weighted | 1.284739078 | 3 | 0.732760084 |
| eqtl-a-ENSG00000172936 | finn-b-F5_DELIRIUM | MR Egger | 2.841829442 | 3 | 0.416660256 |
| eqtl-a-ENSG00000172936 | finn-b-F5_DELIRIUM | Inverse variance weighted | 3.004447202 | 4 | 0.557081448 |

Table S9. Pleiotropy test.

| SYMBOL | id.exposure | id.outcome | egger_intercept | se | pval |
| --- | --- | --- | --- | --- | --- |
| COMT | eqtl-a-ENSG00000093010 | finn-b-F5_DELIRIUM | -0.003075826 | 0.046696132 | 0.953474052 |
| CYP1B1 | eqtl-a-ENSG00000138061 | finn-b-F5_DELIRIUM | 0.007578654 | 0.027272259 | 0.786251427 |
| MYD88 | eqtl-a-ENSG00000172936 | finn-b-F5_DELIRIUM | 0.026494793 | 0.065701691 | 0.713777743 |
